# Cyclopædia of Obstetrics and Gynecology

**Published:** 1887-06

**Authors:** 


### Book Reviews.

---

CYCLOPÆDIA OF OBSTETRICS AND GYNÆCOLOGY. By Dr. A. Charpentier, Adjunct Professor at the Faculty of Medicine, Paris. Translated under the supervision of, and with notes and additions by, Egbert H. Grandin, M. D., Obstetric Surgeon to the New York Maternity Hospital, etc. Vol. I. Anatomy of the Genitals, Menstruation and Fecundation, Normal Pregnancy and Labor. Vol. II. The Pathology of Pregnancy. Vol. III. The Pathology of Labor. New York: Wm. Wood & Co. 1887.

These volumes form a portion of the elaborate work by Charpentier, which appeared in 1882, and which is recognized as one of the classics of medicine. For bringing within the reach of English-speaking readers this valuable collection of the results of wide clinical observation and thorough research into the literature of the subject, the translator has merited the thanks of the profession.

Three volumes, consisting in the aggregate of over twelve hundred pages, devoted to the subject of obstetrics, must of necessity be rich in detail, and such is found to be the case in this instance. Every subject is treated with a completeness which, as far as we know, has no parallel in any other work on obstetrics in the English language. For this reason the work is more valuable as a book of reference than as a hand-book.

Although the obstetrical art is as old as the human race, it has not yet reached its ultimatum. Every year shows progress and improvement. Hence a work upon this subject, which is five years old, must, as a matter of course, be to some extent behind the times. To obviate this objectionable feature, the editor has endeavored, by additions to the text, to bring the work up abreast of the most advanced ideas of the present day. In the main his purpose has been successfully accomplished. Objection might be made, however, to the dogmatic manner in which he has advanced as facts mere theories which are as yet *sub judice*, and which are still opposed by some of the best authorities of the present day. A presentation of arguments and proofs would have carried greater weight than bold assertions. The statement, for instance, that "the catheter should never be passed by touch, but always by sight," is too startling an innovation to the average practitioner to be accepted without the support of substantial reasons. Similarly in regard to Crede's method of delivering the placenta, the use of ergot as routine practice, and the application of the binder, the editor has mistaken his individual opinion for the verdict of the profession. One feels like asking if it is the "gynæcological fashion of speaking *ex cathedra*" which leads him, on page 462, Vol. I, to characterize Charpentier's views as "an error" upon a point which is not yet definitely settled.

We are surprised that the editor has allowed to pass unchallenged the author's statement of his views upon the relation between ovulation and menstruation when he says, "These two functions are considered as one, the last being the external manifestation of the first." In the light of the most recent investigations, such an opinion is hardly tenable, and has few advocates at the present day. The allusion of the editor to "New England, the birthplace of ether," can be ascribed to nothing less than ignorance or prejudice.

These blemishes are but minor ones, however, and serve only to emphasize the general excellence of the work. The chapters upon "Impregnation and Conception," "The Signs and Diagnosis of Pregnancy" and "Maternal Dystocia" are particularly good. The whole work is rich in statistics, introduced to substantiate the views of the author, and we are, therefore, not compelled to accept arbitrary statements of facts and principles. The nationality of the author crops out here and there in his evident antagonism to everything which is of German origin. He also ignores almost entirely the work of American obstetricians.

The mechanical execution is good, the plates and cuts are exceptionally fine and the volumes are of convenient size for handling. Altogether, the work is a valuable addition to obstetrical literature. V. O. H.

